# Association between Serum 25-Hydroxyvitamin D Concentrations and Academic Performance among Adolescent Schoolchildren: A Cross-Sectional Study

**DOI:** 10.3390/nu15214552

**Published:** 2023-10-27

**Authors:** Ahmed A. Hassan, Mustafa I. Elbashir, Abdullah Al-Nafeesah, Ashwaq AlEed, Ishag Adam

**Affiliations:** 1 Faculty of Medicine, University of Khartoum, Khartoum 11115, Sudan; aa801181@gmail.com (A.A.H.);; 2 Department of Pediatrics, Unaizah College of Medicine and Medical Sciences, Qassim University, Unaizah 51911, Saudi Arabia; a.alnafeesah@qu.edu.sa; 3 Department of Pediatrics, College of Medicine, Qassim University, Buraydah 52571, Saudi Arabia; 4 Department of Obstetrics and Gynecology, Unaizah College of Medicine and Medical Sciences, Qassim University, Unaizah 51911, Saudi Arabia; ishagadam@hotmail.com

**Keywords:** female, schoolchildren, age, vitamin D, 25(OH)D, adolescent, academic performance, Sudan

## Abstract

The level of association between 25-hydroxyvitamin D (25[OH]D) levels and students’ academic performance has not yet been established. The current study aimed to investigate the association between serum 25(OH)D levels and academic performance among schoolchildren in Sudan. A cross-sectional study was conducted among schoolchildren during the 2021/2022 academic year from four randomly selected schools in Almatamah, River Nile State, northern Sudan. Sociodemographic data were collected using a questionnaire. Anthropometric measurements were performed in accordance with standard procedures. Academic performance was obtained from school records. Serum 25(OH)D levels were measured, and regression (multiple linear regression and multivariate logistic) analyses were performed. A total of 241 participants were enrolled in this study, of whom 129 (53.5%) were female. The mean standard deviation (SD) of the participants’ ages was 15 ± 1.6 years. In multiple linear regression tests, being female, age, employment, and serum 25(OH)D level were positively associated with academic performance. The average overall academic score was 33.74%. Of the 241 participants, 95 (39.4%) and 149 (61.6%) had good and poor academic performances, respectively. In multivariable logistic regressions, age and 25(OH)D level were inversely associated with poor academic performance and vitamin D deficiency was associated with poor performance. The current study revealed a positive association between 25(OH)D levels and adolescents’ academic performance. Effective interventional programs are needed to maintain sufficient vitamin D levels during childhood and adolescence and, as a consequence, to improve academic performance.

## 1. Introduction

Several studies across multiple countries have reported the influence of child health and nutrition on education and employment [1,2]. There is a rapidly declining trend in students’ academic performance, especially in African countries [3,4,5]. For example, in Africa, various educational challenges exist, such as the poor quality of education and lack of resources [4,5]. Several factors, such as gender [3,6], high socioeconomic status [7], school type (public or private) [3], and anthropometric measurements, including body mass index [6,7], anemia [8,9,10], and vitamin D deficiency (defined as 25-hydroxyvitamin D [25(OH)D] <20 ng/mL) [11,12], were all reported to be associated with academic performance. In addition, several diseases are reported to be associated with vitamin D deficiency, such as headaches [13]; several psychological disorders, including stress, depression, and anxiety [14]; and sleep disorders, including insomnia and obstructive sleep apnea [15]; neuropsychiatric disorders; and autoimmune diseases [16]. Such diseases could heavily impact the students’ concentration power and school attendance, which would collectively result in poor academic performance. In recent years, more attention has been given to the influence of vitamin D status on students’ academic performance [11,12]. However, the results of the few existing data are inconsistent: while some studies have found a significant association between vitamin D status and students’ academic performance [11,12], others have found no such association [17]. Therefore, such data inconsistency regarding the association between 25(OH)D level and students’ academic performance necessitates further research, especially in understudied settings with limited resources. Moreover, nutrition, education, and health are key components in the Sustainable Development Goals (SDGs) [18], and the heavy burdens of vitamin D deficiency on children and adolescents’ health, especially in Africa, is remarkable [19,20]. Thus, the practical steps to improve students’ academic performance necessitate a thorough understanding of the local context. To achieve this, first, the current students’ performance and its associated factors, including 25(OH)D level, need to be investigated. Then, appropriate measurements can be implemented by all involved parties to improve the current situation.

Although the impacts of certain diseases, such as type 1 diabetes mellitus and sleeping disorders, on the academic performance of schoolchildren and medical students [21,22] were studied in Sudan, 25(OH)D level has not yet been explored in similar settings. However, a high prevalence of vitamin D deficiency and its complications across the country regions in different groups, including children and women, were reported in Sudan [23,24].

On the other hand, despite the Government of Sudan’s efforts to improve education, poor academic performance was reported among adolescent students [18,25]. However, the association between vitamin D status and students’ academic performance has not been investigated in Sudan. Hence, such an association needs to be explored, aiming to address both these issues (25(OH)D level and academic performance). Thus, the current study aimed to investigate the association between serum 25(OH)D levels and academic performance among schoolchildren in North Sudan.

## 2. Materials and Methods

### 2.1. Study Area

There are 18 states in Sudan, and the River Nile is one of them. Based on the last census (2008 census), River Nile State had a total population of 1,120,441 [26]. River Nile State has seven localities. (The locality is defined by the Sudan Government as the lowest administrative unit.) More details of the studied area can be found in our previous published work [27].

### 2.2. Study Population and Design

This cross-sectional study was conducted between July and September 2022 in four primary and secondary schools in the Wad Hamid district, Almatamah locality, River Nile State, northern Sudan. Almatamah is the neighboring locality of Khartoum State and is about 150 km from Khartoum City, the capital of Sudan. This study followed the reporting of observational studies in epidemiology (STROBE) guidelines [28].

**Figure 1:**
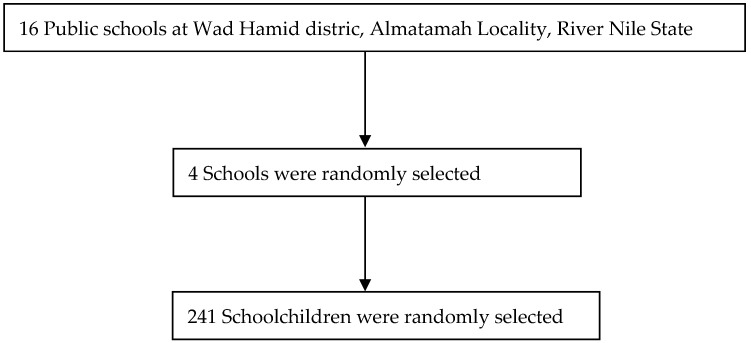
Flow chart of the sample selection of schoolchildren during the 2021/2022 academic year in Almatamah, River Nile State, northern Sudan.

Initially, from the seven localities, one locality (Almatamah) was randomly selected using simple random sampling. Then, Wad Hamid was selected randomly from the three districts of the Almatamah locality. From a total of 16 schools in the Wad Hamid district, four public schools for girls and boys were selected randomly. The total list of students in these four schools was obtained from the schools’ managers. Then, certain numbers of students from each school were selected randomly from the provided list based on each school’s total number of students to reach the desired sample size (n = 241) (Figure 1). The investigators trained four research assistants to collect the data. After signing an informed consent form, the questionnaires (from each student) were completed by the research assistants. This questionnaire was based on similar previous studies [6,7,10,11,12,29]. The questionnaire contained questions about sociodemographic data, such as gender (male or female), age in years, and parents’ education (secondary level and ≥ secondary) and occupation status.

Students’ academic performance (the overall score) was assessed based on the last marks awarded to them in the school examinations (the total score was 100%). Furthermore, academic performance was categorized based on the mean into less than the mean score (poor performance) versus more than the mean score (good performance).

### 2.3. The Inclusion and Exclusion Criteria

The inclusion criteria included the following: Any student from the selected schools who signed an informed consent, was between 10 and 19 years old based on the World Health Organization (WHO) definition of adolescents [30], and appeared healthy was eligible to participate in the study.

Although all students from the selected schools were approached to participate in the study, those who refused to give consent, with learning disabilities or chronic diseases, such as respiratory, cardiac, hematological, and immunological diseases, were excluded from the study.

### 2.4. Procedures

The students’ weight was measured in kilograms (kg) using standard procedures (well-calibrated scales adjusted to zero before each measurement). The students stood with minimal movement, with their hands by their sides. Moreover, shoes and excess clothing were removed. Then, height was measured in centimeters (cm) after the students were made to stand straight with their feet together and their backs against the wall. Moreover, the BMI for the age Z-score was determined according to the WHO standards [31].

### 2.5. Processing of Blood Samples

Five milliliters of blood were collected from the cubital vein into a plain tube and allowed to clot at room temperature. An automated hematology analyzer was used to measure the hemoglobin level according to the manufacturer’s instructions (Sysmex KX-21, Kobe, Japan) [32]. Furthermore, we performed the assay of 25(OH)D using the enzyme-linked immunosorbent assay (fully automatable 450 nm, reference wavelength between 620 and 650 nm) following the manufacturer’s instructions (Euroimmun, Lubeck, Germany). Manufacturer quality control measures and 6 levels of standard solutions (calibrator) set between 0 and 120 ng/mL were applied for each assay. More details about this assay are reported in our previously published work [33].

In the present study, the definition of anemia in adolescents was based on the WHO definition (i.e., the age- and sex-specific cutoff levels of individual hemoglobin levels of <12 g/dL for females and <13 g/dL for males), and anemia was considered severe if hemoglobin was <8 g/dL [34]. The sample was considered vitamin D deficient if the serum 25(OH)D level was <20 ng/mL and the normal 25(OH)D status was ≥20 ng/mL [35]. Furthermore, 25(OH)D level is subcategorized into severe vitamin D deficiency (a serum level < 10 ng/mL), vitamin D deficiency (a serum level of <20 ng/mL), and vitamin D insufficiency (a serum level < 30 ng/mL). This cutoff point was adopted in this study based on previous similar studies which used the same cutoff point among schoolchildren for vitamin D deficiency [14,36,37].

### 2.6. Sample Size Calculation

As previously mentioned [38], a sample of 241 students was computed to have a significant minimum difference in the correlations (r = 0.18) between the 25(OH)D level and academic performance. This sample size of 241 students was calculated to detect a difference of 5% at α = 0.05 with a power of 80%.

### 2.7. Statistical Analysis

The collected data were entered into a computer using IBM Statistical Product and Service Solutions (SPSS) for Windows (version 22.0; SPSS Inc., New York, NY, USA). Continuous data were evaluated for normality using the Shapiro–Wilk test, which revealed normally distributed age and non-normally distributed 25(OH)D and hemoglobin. The non-normally distributed data were expressed as a median (interquartile range, IQR) and compared between the two groups using the Mann–Whitney *U* test. Normally distributed continuous data were expressed as a mean (standard deviation, SD) and were compared between the two groups using a *t*-test. Categorical data were expressed as frequencies (%) and analyzed using the chi-squared test. Both multiple linear and multivariate regressions were performed to evaluate academic performance using continuous variables for linear regression and poor as less than mean versus good as more than mean score for multivariate analysis. The independent variables were gender, age, parents’ education and occupation (the BMI for age Z-score), and 25(OH)D level/vitamin D status (entered one by one in the model). For both, linear and multivariate analysis, variables in the univariate analysis with a *p*-value of <0.20 were entered into the linear and multivariate logistic regression to adjust for covariates. The adjusted odds ratios (AORs), 95% confidence intervals (CIs), coefficients, and standard errors were calculated as applied. A two-sided *p*-value of <0.05 was considered statistically significant.

## 3. Results

### 3.1. General Characteristics

A total of 241 participants were enrolled in this study. Of the total 241 participants, 129 (53.5%) were female and the remaining 112 (46.5%) were male. The mean (SD) of the participants’ age was 15 ± 1.6 years. Of the total 241 participants, 156 (64.7%) mothers had education ≥ secondary level, and the remaining 85 (35.3%) had their education < secondary level. Moreover, out of the total, 162 (67.2%) fathers had their education ≥ secondary level, and the remaining 79 (32.8%) had their education < secondary level. A majority of the mothers (211, 87.6%) were housewives. Of the total 241, 98 (40.7%) fathers were farmers and the remaining 143 (59.3%) were laborers. The median (IQR) of the BMI Z-score was −0.46(−1.59–0.50). Fifty-seven (23.7%) participants were anemic. The median (IQR) of the participants’ serum 25(OH)D was 13.0 (12.3–14.0) ng/mL, and 117 (48.5%) participants had vitamin D deficiency (Table 1). Of the total 241, 24.1% had severe vitamin D deficiency (<10 ng/mL), 48.5% had vitamin D deficiency (<20 ng/mL), 70.5% had vitamin D insufficiency (<30 ng/mL), and 29.5% had vitamin D sufficiency (≥30 ng/mL).

### 3.2. Factors Associated with Academic Performance in %

**Figure 2:**
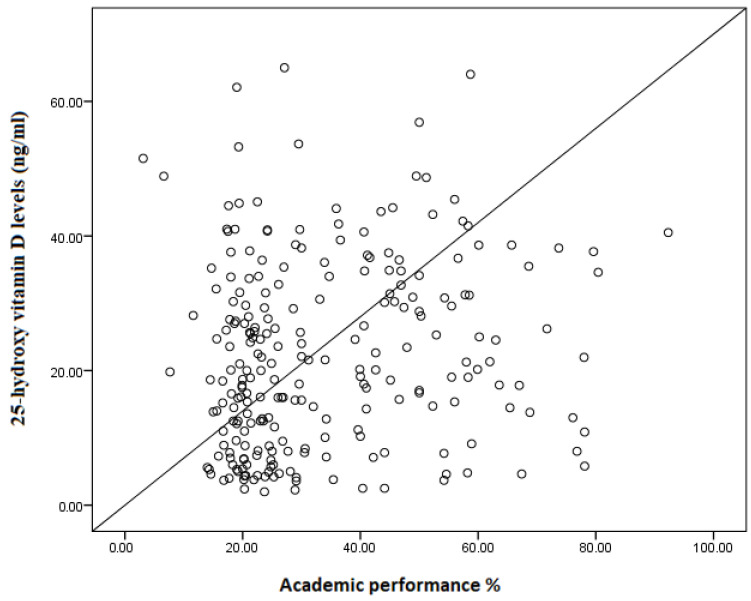
Correlation between 25-hydroxy vitamin D levels (ng/mL) and academic performance (%) among schoolchildren in northern Sudan, 2022 (n = 241), (coefficient = 0.20, *p* = 0.010).

In the multiple linear regression, while there was a positive association between age (coefficient = 3.23, *p* < 0.001), being female (coefficient = 7.62, *p* = 0.002), employment (coefficient = 6.27, *p* = 0.049), serum 25(OH)D level (coefficient = 0.20, *p* = 0.010), and academic performance, there was no association between mother’s education, father’s education, occupation, BMI Z-score, anemia, and academic performance (Table 2, Figure 2).

### 3.3. Factors Associated with Academic Performance (Good vs. Poor)

**Figure 3:**
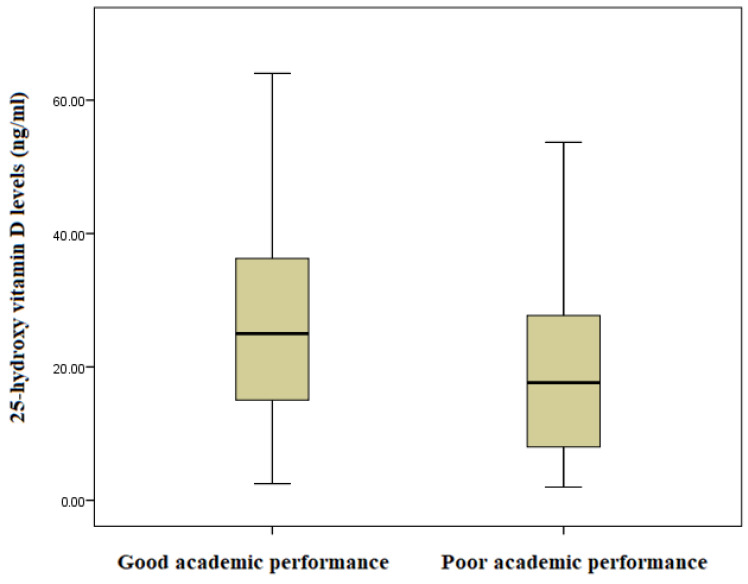
Comparison of 25-hydroxy vitamin D levels (ng/mL) in good and poor academic performance groups among schoolchildren in northern Sudan, 2022 (n = 241). Poor academic performance compared to adolescents with good academic performance (17.65 [8.0–27.78] vs. 25.0 [14.72–36.44] ng/mL, *p* = 0.001).

The average overall academic score was 33.74%. Of the 241 enrolled participants, 95 (39.4%) and 149 (61.6%) had good (more than average) and poor (less than average) academic performances, respectively. The median (IQR) of serum 25(OH)D was significantly lower among adolescents with poor academic performance compared to adolescents with good academic performance (17.65 [8.0–27.78] vs. 25.0 [14.72–36.44], *p* = 0.001) (Figure 3). The levels of vitamin D deficiency among participants with poor and good academic performance were 81 (55.5%) and 36 (37.9%), *p* = 0.008, respectively.

In univariate logistic regression, being male, the mother’s education and occupation, father’s education and occupation, BMI for age Z-score, and anemia were not associated with poor academic performance; the student’s age and serum 25(OH)D level were associated with poor academic performance (Table 3).

In multivariable logistic regressions, while there was no association between gender, father’s education, BMI for age Z-score, academic performance, and age (AOR = 0.59, 95% CI 0.48–0.72), 25(OH)D levels (AOR = 0.97, 95% CI 0.95–0.99) were associated with decreased odds of poor academic performance, and vitamin D deficiency (AOR = 2.18, 95% CI 1.20–3.96) was associated with increased odds of poor academic performance (Table 4).

## 4. Discussion

The key findings of the present study were that in this rural community, 61.6% of the enrolled students had poor academic performance, and serum 25(OH)D level was positively associated with students’ academic performance. The poor academic performance of students observed in this study supports the United Nations Children’s Fund (UNICEF) report, which stated that Sudan remained far from achieving the SDG 4, which aims to “ensure inclusive and equitable quality education and promote lifelong learning opportunities for all” [25]. According to UNICEF, several factors, including poverty, geographical disparities, gender inequities, disability, conflict, and displacement, have been reported to contribute to poor access, retention, and learning outcomes in basic education in Sudan [25].

The present study showed that serum 25(OH)D level was positively associated with students’ academic performance, and students with vitamin D deficiency were more than two times at risk of having poor academic performance. This result is similar to previous studies [11,12,39]. For example, in Saudi Arabia, a study that included 213 medical and health science students showed that 25(OH)D level had a significant association with the overall performance of the students [12]. Another comparative cross-sectional study that included 480 university students revealed that a high proportion of students with vitamin D deficiency attained a low or moderate grade point average (GPA) compared to the control cohort [39]. Additionally, in India, a clinic-based cross-sectional study including 62 healthy adolescents (aged 10–19 years) presenting with school reports of recent onset poor scholastic performance revealed the highest prevalence of vitamin D deficiency in adolescents with scholastic backwardness [11].

In contrast, other studies have revealed that serum 25(OH)D concentrations are not associated with academic performance in adolescents [17,40]. For example, in Kuwait, a school-based cross-sectional study that included 1370 adolescents concluded that 25(OH)D was not associated with cognitive function or academic performance in adolescents [17].

The exact mechanism underlying vitamin D’s influence on academic performance is not well understood. However, based on the existing literature, the influence of vitamin D status on academic performance could be attributed to several reasons, including the impact of vitamin D deficiency in children and adolescents on mental health and psychological wellbeing [39], increasing risk of headaches in the pediatric and adult population [13], vulnerability to respiratory tract infections [41], sleep disorders such as insomnia and obstructive sleep apnea, restless legs syndrome [15], tiredness, low mood, and chronic fatigue [42]. Such health conditions can contribute to students’ absence from attending classes, lack of attention during class hours and, as a result, poor academic performance. For example, Turner et al. in their cross-sectional analysis, which included 34 ,403 children in the United States, reported that children with headaches were more likely to experience poor school attendance, poor academic performance, and lower quality of life in comparison with children without headaches [43].

To minimize the risk of vitamin D deficiency and its complications, certain interventions can be initiated. For example, school-based and media-based campaigns can be a good approach to raising awareness about the importance of vitamin D and its sources (diet and sun exposure) and risks [41,44]. For example, the local authority needs to check the status of school’s implementation of nutritional school programs and to act accordingly (i.e., implementing new programs or improving the existing ones). It is worth mentioning that there was low awareness of the impact of vitamin D deficiency on academic performance, even among medical students [12,45].

In the current study, BMI for age Z-score was not associated with academic performance. In line with this result, a study from Ethiopia showed that BMI for age Z-score was not associated with academic performance among 131 schoolchildren (age 8–11 years) [7]. In contrast, another study showed a positive association between BMI for age Z-score and academic performance [6]. Specifically, in Ethiopia, a study showed an increase in the mean mark score of students by 1.89 for a unit increase in BMI for age Z-score [6]. Such variation in the influence of BMI on academic performance may indicate a lower reliability of BMI in assessing total body fat [46]. In addition, some studies have shown a reciprocal association between leptin and 25(OH)D levels among adolescents [47]. Moreover, high levels of leptin are associated with poorer academic performance [48].

In the current study, in linear regression analysis, being female contributed to higher scores compared to males (coefficient = 7.62, *p* = 0.002). In multivariate analysis, on the other hand, gender was not associated with academic performance. In our neighboring country, Ethiopia, Katiso et al. reported that female adolescents decreased their mean mark score by 2.63 times compared to male adolescents [6]. It seems that gender differences in adolescents’ academic performance rely on multiple factors, including sociocultural factors, behaviour, and even safety in rural remote areas [6,25,49,50].

In the current study, there was no association between parental education and occupation status and poor academic performance; however, students of employed mothers were more likely to have high academic scores compared to their counterparts. Several studies have reported the impact of parental education and occupation on adolescents’ academic performance [51,52]. For example, Tadese et al. found no impact of parental education or occupation on academic performance among university students in Ethiopia [51]. In our context, the lack of parental education and occupation status on adolescents’ academic performance could be explained by the low quality of education provided to the parents as well as the similarity of wages of farmers and laborers in this rural community setting as a consequence of similar socioeconomic status. Moreover, socioeconomic status might enhance the effect of relative age on academic performance [53]. Parental education and occupational status need to be reflected in parental involvement in school to result in good children’s academic outcomes, regardless of parental education and occupational status [54].

In the current study, anemia was not associated with academic performance. Similarly, in Mexico, Vega-Franco et al. showed no association between hemoglobin levels and intelligence test results [55]. In contrast, other studies have found an association between academic performance and anemia in adolescents [9,10]. The effects of hemoglobin levels on cognitive function among adolescents could be attributed to the different types of anemia [56], which was not investigated in the current study.

The present study showed an association between increased age and improved academic performance. This is similar to previous studies that have addressed the importance of age in academic performance [53,57]. This positive association between older age and improved academic performance among adolescents could indicate the cumulative experiences of learning skills, the relatively low prevalence of vitamin D deficiency among older students, and the trend of improving academic performance by age. In Kuwait, a study including 199 schoolchildren showed that being younger (≤8.5 years) was a significant risk factor for 25(OH)D deficiency [37]. Kuang et al. indicated that lead exposure can have adverse health effects on children’s erythrocyte parameters, BMI, and poor academic performance [58], i.e., an increase in age and, as a consequence, increase in BMI might decrease lead concentrations in the body, which lead to improving academic performance. Therefore, measuring both serum 25(OH)D level and serum lead level is advisable in future studies

Our results should be compared cautiously with findings to the contrary in previous studies. First, the present study was conducted in a rural community setting with only public schools, and different academic performances between setting (urban versus rural) and school type (public versus private) were reported [3,18]. Second, differences in the heterogeneity of participants’ baseline sociodemographic characteristics, such as age, should be taken into account, as in our study (10–19 years). Third, there were differences in cutoff points for vitamin D deficiency; however, in this study, the commonly used one was adopted, i.e., the serum 25(OH)D level of <20 ng/mL [35].

Although the present study adds novel insights to the few existing research papers on the association between vitamin D status and academic performance [11,12,17], especially from an understudied country (Sudan), it has some limitations that need to be acknowledged. Due to the nature of the present cross-sectional study, it is difficult to establish causality between vitamin D deficiency and academic performance. Thus, a longitudinal study will provide more information on such an association. The current study will also not provide nationally representative data on vitamin D deficiency among Sudanese adolescents because it covered only one geographical region (northern Sudan). Furthermore, the present study did not collect other information in comparison with other studies, such as lifestyle, including physical activity [59,60], dietary intake [39], and heavy metal (e.g., lead) exposure [58]. In the current study, we assumed a similar exposure to sunlight among the studied adolescents since the studied area is a rural area. Furthermore, the current study did not gather information about the eating habits of the adolescents since sun exposure is not the only source to receive vitamin D from as dietary sources also play a key role in vitamin D status, nor did the current study gather any information about the diet history from the participants. Therefore, such information is essential to be considered in future research.

## 5. Conclusions

The current study revealed a positive association between vitamin D status and adolescents’ academic performance. Effective interventional programs are needed to maintain sufficient vitamin D levels during childhood and adolescence and, as a consequence, to improve academic performance.

## Figures and Tables

**Table 1:** General characteristics of schoolchildren in northern Sudan, 2022 (n = 241).

Variable		
		Mean (standard deviation)
Age, years		15.1 (1.6)
		Median (interquartile range)
Body mass index for age Z-score		−0.46 (−1.59–0.50)
25-hydroxy vitamin D concentration, ng/mL		20.10 (10.56–31.87)
		Frequency (proportions)
Gender	Female	129 (53.5)
	Male	112 (46.5)
Mother’s education level	≥secondary	156 (64.7)
	<secondary	85 (35.3)
Mother’s occupation	Housewife	211 (87.6)
	Employed	30 (12.4)
Father’s occupation	Laborer	143 (59.3)
	Farmer	98 (40.7)
Father’s education	≥secondary	162 (67.2)
	<secondary	79 (32.8)
Vitamin D status	Normal ≥ 20 ng/mL	124 (51.5)
	Deficient < 20 ng/ml	117 (48.5)
Anemia	No	184 (76.3)
	Yes	57 (23.7)

**Table 2:** Multiple linear regression analysis of the factors associated with academic performance among schoolchildren in northern Sudan, 2022 (n = 241).

Variable		Academic Performance Score in %	
		Coefficient (Standard Error)	*p* Value
Age, years		3.23 (0.67)	<0.001
Body mass index for age Z-score		1.40 (0.78)	0.075
25-hydroxy vitamin concentration (ng/mL)		0.20 (0.08)	0.010
Anemia		−0.491 (2.45)	0.841
Gender	Male	Reference	
	Female	7.62 (2.37)	0.002
Mother’s education	≥secondary	Reference	
	<secondary	−0.77 (2.35)	0.743
Mother’s occupation	Housewife	Reference	
	Employed	6.27 (3.13)	0.049
Father’s education	≥secondary	Reference	
	<secondary	−3.47 (2.51)	0.167
Father’s occupation	Laborer	Reference	
	Farmer	−0.73 (2.10)	0.730
Anemia	No	Reference	
	Yes	−0.491 (2.45)	0.841

**Table 3:** Univariate analysis of the factors associated with poor academic performance among schoolchildren in northern Sudan, 2022 (n = 241).

Variable		Poor Academic Performance (Score < 3.74%) (n = 0.146)	Good Academic Performance (Score ≥ 33.74%) (n = 95)	Odds Ratio	95% Confidence Interval	*p* Value
		Mean (standard deviation)				
Age, years		14.6 (1.6)	15.8 (1.3)	0.59	0.49–0.72	<0.001
		Median (interquartile range)				
Body mass index for age Z-score		−0.55 (−1.66–0.37)	−0.30 (−1.36–0.74)	0.86	0.71–1.03	0.107
25-hydroxy vitamin D concentration (ng/mL)		17.65 (8.0–27.78)	25.0 (14.72–36.44)	0.98	0.96–0.99	0.008
		Frequency (proportions)				
Gender	Female	73 (50.0)	56 (58.9)	Reference		0.174
	Male	73 (50.0)	39 (41.1)	1.44	0.85–2.42	
Mother’s education level	≥secondary	95 (65.1)	61 (64.2)	Reference		0.892
	<secondary	51 (34.9)	34 (35.8)	1.04	0.61–1.79	
Mother’s occupation	Housewife	131 (89.7)	80 (84.2)	Reference		0.208
	Employed	15 (10.3)	15 (15.8)	0.61	0.28–1.32	
Father’s occupation	Laborer	64 (43.8)	34 (35.8)	Reference		0.215
	Farmer	82 (56.2)	61 (64.2)	1.40	0.82–2.38	
Father’s education	≥secondary	53 (36.3)	69 (72.6)	Reference		0.150
	<secondary	93 (63.7)	26 (27.4)	1.52	0.86–2.63	
vitamin D status	Normal ≥ 20	65 (44.5)	59 (62.1)	Reference		0.008
	Deficient < 20	81 (55.5)	36 (37.9)	2.04	1.21–3.46	
Anemia	Non-anemic	108 (74.0)	76 (80.0)	Reference		0.283
	Yes	38 (26.0)	19 (20.0)	1.41 (0.75–2.63)		

**Table 4:** Multivariate analyses of the factors associated with poor academic performance among schoolchildren in northern Sudan, 2022 (n = 241).

Variable		Adjusted Odds Ratio (95% Confidence Interval)	*p* Value
Age, years		0.59 (0.48–0.72)	<0.001
Body mass index for age Z-score		0.86 (0.70–1.05)	0.139
25-hydroxy vitamin concentration (ng/mL) *		0.97 (0.95–0.99)	0.016
Gender	Female	Reference	
	Male	1.60 (0.88–2.92)	0.126
Father’s education	≥secondary	Reference	
	<secondary	1.64 (0.88–3.03	0.121
Vitamin D status *	Normal ≥ 20 ng/mL	Reference	
	Deficient < 20 ng/mL	2.18 (1.20 –3.96)	0.011

* Were entered one by one.

## Data Availability

The data will be available from the corresponding author upon reasonable request.
